# What are the most important unanswered research questions on rapid review methodology? A James Lind Alliance research methodology Priority Setting Partnership: the Priority III study protocol

**DOI:** 10.12688/hrbopenres.13321.2

**Published:** 2021-11-18

**Authors:** Claire Beecher, Elaine Toomey, Beccy Maeso, Caroline Whiting, Derek C. Stewart, Andrew Worrall, Jim Elliott, Maureen Smith, Theresa Tierney, Bronagh Blackwood, Teresa Maguire, Melissa Kampman, Benny Ling, Christopher Gravel, Catherine Gill, Patricia Healy, Catherine Houghton, Andrew Booth, Chantelle Garritty, James Thomas, Andrea C. Tricco, Nikita N. Burke, Ciara Keenan, Matthew Westmore, Declan Devane

**Affiliations:** 1 Evidence Synthesis Ireland and Cochrane Ireland, Galway, Ireland; 2 School of Nursing and Midwifery, National University of Ireland Galway, Galway, Ireland; 3 School of Allied Health, University of Limerick, Limerick, Ireland; 4 James Lind Alliance, University of Southampton, Southampton, UK; 5 Honorary Professor, National University of Ireland Galway, Galway, Ireland; 6 Public co-author, Evidence Synthesis Ireland, Galway, Ireland; 7 Public co-author, Staffordshire, UK; 8 Public co-author, Cochrane Consumer Network Executive, Ottawa, Canada; 9 Patient Partner, HRB Primary Care Clinical Trials Network Ireland, Galway, Ireland; 10 Wellcome-Wolfson Institute of Experimental Medicine, Queen's University Belfast, Belfast, UK; 11 Health Research Board, Dublin, Ireland; 12 Health Canada, Ottawa, Canada; 13 School of Epidemiology and Public Health, University of Ottawa, Ottawa, Canada; 14 School of Health And Related Research (ScHARR), University of Sheffield, Sheffield, UK; 15 Public Health Agency of Canada, Ottawa, Canada; 16 EPPI-Centre, UCL Social Research Institute, University College London, London, UK; 17 Knowledge Translation Program, Li Ka Shing Knowledge Institute, St. Michael’s Hospital, Unity Health Toronto, Toronto, Ontario, Canada; 18 Epidemiology Division and Institute for Health Policy, Management, and Evaluation, Dalla Lana School of Public Health, University of Toronto, Toronto, Ontario, Canada; 19 Queen's Collaboration for Health Care Quality JBI Centre of Excellence, School of Nursing, Queen’s University, Kingston, Ontario, Canada; 20 Campbell UK & Ireland, Queen's University Belfast, Belfast, UK; 21 University of Southampton, Southampton, UK; 22 Health Research Board- Trials Methodology Research Network, Galway, Ireland

**Keywords:** Rapid review, systematic review, methodology, evidence synthesis, Priority setting partnership, PPI

## Abstract

**Background: **The value of rapid reviews in informing health care decisions is more evident since the onset of the coronavirus disease 2019 (COVID-19) pandemic. While systematic reviews can be completed rapidly, rapid reviews are usually a type of evidence synthesis in which components of the systematic review process may be simplified or omitted to produce information more efficiently within constraints of time, expertise, funding or any combination thereof. There is an absence of high-quality evidence underpinning some decisions about how we plan, do and share rapid reviews. We will conduct a modified James Lind Alliance Priority Setting Partnership to determine the top 10 unanswered research questions about how we plan, do and share rapid reviews in collaboration with patients, public, reviewers, researchers, clinicians, policymakers and funders.

**Methods: **An international steering group consisting of key stakeholder perspectives (patients, the public, reviewers, researchers, clinicians, policymakers and funders) will facilitate broad reach, recruitment and participation across stakeholder groups. An initial online survey will identify stakeholders’ perceptions of research uncertainties about how we plan, do and share rapid reviews. Responses will be categorised to generate a long list of questions. The list will be checked against systematic reviews published within the past three years to identify if the question is unanswered. A second online stakeholder survey will rank the long list in order of priority. Finally, a virtual consensus workshop of key stakeholders will agree on the top 10 unanswered questions.

**Discussion: **Research prioritisation is an important means for minimising research waste and ensuring that research resources are targeted towards answering the most important questions. Identifying the top 10 rapid review methodology research priorities will help target research to improve how we plan, do and share rapid reviews and ultimately enhance the use of high-quality synthesised evidence to inform health care policy and practice.

## Introduction

Systematic reviews summarise existing research and use structured and ‘explicit methods to identify, select and critically appraise relevant studies, and collect and analyse data from these studies’ ^1^. Systematic reviews, therefore, provide a robust synthesis of existing research for a given topic and are typically seen as the cornerstone of research evidence for informing health policy and practice decision-making. However, systematic reviews require significant resources (time, expertise, funding) to conduct and can take up to two years to complete ^2^. While full systematic reviews can, and have been, completed rapidly, rapid reviews have emerged more commonly as a form of evidence synthesis in which certain steps of the systematic review process are omitted or simplified to produce information more efficiently within limited resources to inform healthcare decisions ^2, 3^.

Evidence suggests that rapid reviews are increasingly being commissioned, and the results used to inform decision making by policymakers and funders as a more resource efficient alternative to conventional systematic reviews ^4, 5^. The coronavirus disease 2019 (COVID-19) pandemic has further increased the demand for rapid reviews due to the need to provide time-sensitive information to decision-makers ^6^.

Although growing demand has led to a steady rise in rapid review publications generally since 2010 ^3, 7^, albeit often poorly reported with many important details not included ^8^, there are challenges in identifying rapid reviews commissioned specifically by organisations as they are often not published in peer review journals ^3^. This may, in part, be due to the suggestion that commissioned rapid reviews are considered more relevant in the context of narrow questions that address a specific question that may be of relevance to that commissioning body alone, rather than broader questions of wider relevance that may require the focus of a conventional systematic review ^5, 9^. 

Despite an evident rise in the commissioning and conduct of rapid reviews, there is an absence of high-quality evidence underpinning some decisions about how they are planned, done and the findings shared. There is also limited evidence on the relative impact of different simplifications or omissions, for example, restricting searches to published material or by date/language or conducting single reviewer screening or data extraction ^10, 11^, on the validity and application of findings ^3^ commonly used in rapid reviews. Furthermore, there is no universally accepted definition of a rapid review ^3, 6, 12, 13^ and debate exists regarding the use of the word ‘rapid’, with several different synonyms previously identified within the literature ^7, 10^. These debates have been further complicated in the era of the COVID-19 pandemic where it has been highlighted that rapid reviews cannot be categorised simply based on the time taken to complete a review given that well-resourced conventional systematic reviews have been conducted to a high standard, rapidly.

Overall, rapid reviews are ‘poorly understood, ill-defined diverse methodologies supported by limited published evidence’ ^3^, with a need for further research to establish a robust methodological evidence base ^14^. Without such an evidence base, the validity, appropriateness and usefulness of rapid reviews are undermined ^2^. 

Research prioritisation plays a key role in minimising research waste by ensuring that research resources are targeted towards questions of the most potential benefit ^15^. Research prioritisation involves identifying, prioritising and obtaining consensus on research needs and questions that are relevant and important to all relevant stakeholders for that topic ^16^. The James Lind Alliance (JLA) is a non-profit organisation that brings multiple stakeholders, including patients, carers and clinicians, together in an equal, transparent and evidence-based Priority Setting Partnership (PSP) to determine the most important evidence uncertainties or unanswered research questions. Although commonly focused on knowledge gaps surrounding the effects of treatments, the approach has been broadly applied to other areas such as diagnosis, prevention, and more recently, to identify methodology uncertainties in recruitment and retention within clinical trials ^17, 18^.

This *Priority III PSP* aims to identify the top 10 unanswered research questions about how we plan, do and share rapid reviews, as identified and prioritised by contributors drawn from key stakeholder groups, including patients, the public, reviewers, researchers, clinicians, policymakers and funders. Evidence Synthesis Ireland is conducting the *Priority III PSP* in collaboration with the JLA. Evidence Synthesis Ireland is an all-Ireland initiative funded by the Health Research Board and the Health and Social Care, Research and Development Division of the Public Health Agency in Northern Ireland. It aims to make evidence syntheses better designed, conducted, reported, and more usable within health care policy and clinical practice decision-making by patients, the public, health care institutions and policymakers, clinicians and researchers.

## Protocol

### Ethical approval

Ethical approval was granted by the National University of Ireland Galway Research Ethics Committee (reference: 20-Apr-02).

### James Lind Alliance PSP process

The study employs a PSP based on the methods of the JLA and will be reported following the REporting guideline for PRIority SEtting of health research (REPRISE) criteria ^16^. 

Following the JLA Guidebook for PSPs ^19^, this PSP will take place in seven stages and build on modified JLA guidance ^17, 18^ and previous PSPs in recruitment and retention within clinical trials ^17, 18^. 

The seven stages of the project are: establishing the steering group, identifying and inviting potential partners, gathering uncertainties, data processing and verifying uncertainties, interim priority setting; final prioritisation workshop; and disseminating findings. 

This protocol was submitted prior to the consensus workshops and published after completion of the final prioritisation workshop. There are several factors that contributed to the late submission of the protocol. We had to balance the evolving nature of the PSP processes and having sufficient clarity on processes for inclusion in a protocol. We also encountered delays due to useful discussions with F1000/ HRB Open Research on affiliation of public partner authors and technical problems with submission attempts prior to the consensus workshop.

***Establishing the Steering Group (6 months-January 2020).*** The Priority III PSP will be led and managed by an international Steering Group who will coordinate, oversee and guide the PSP activities. The primary roles of the Steering Group will be to discuss and agree on the scope and remit of the project, enable access and reach to key stakeholder groups, contribute intellectually towards the study methods and interpretation, and ensure that all perspectives are captured and meaningfully included throughout.

The Steering Group will also determine decisions about the processes used throughout as the project progresses. Membership of the Steering Group will include individuals and representatives from organisations, including patients and the public, reviewers, researchers, clinicians, policymakers and funders. The Steering Group will comprise of up 25 members across stakeholder groups. In line with the international relevance of the PSP topic, steering group members will be drawn from a wide geographical spread. Potential members will be approached to participate via email. Patient and public participants will be paid for their time spent on Steering Group activities. Other participants (researchers/ reviewers, clinicians, policy makers, funders) will not be paid.

***Identifying and inviting potential partners (7 months- April 2020).*** Partners are organisations, groups and individuals impacted by how rapid reviews are planned, done and shared. They will commit to supporting the PSP, promoting the process and encouraging their represented groups or members to participate ^19^. Steering Group members will identify and engage additional appropriate partners through a process of peer knowledge and consultation, using the Steering Group members’ respective networks. Organisations and individuals who can reach and advocate for key stakeholder groups will be invited to participate with the PSP as partners *.* We have not set a minimum or maximum number of partners we would like to recruit. Partners will be organisations or groups representative of diverse stakeholder perspectives (patients and the public, reviewers, researchers, clinicians, policymakers and funders) who will commit to supporting, participating and promoting the PSP among their stakeholder groups. As far as possible, the partners involved will seek to represent the interests of all stakeholders. All partners will be asked to confirm that they agree to support the PSP.

***Gathering uncertainties (1 month-October 2020).*** The Rapid Review Methodology PSP will gather uncertainties from patients and the public, reviewers, researchers, clinicians, policymakers and funders. This will be undertaken by conducting an initial survey with all relevant stakeholders. The survey will identify unanswered questions or uncertainties about how we plan, do and share rapid reviews. The survey will be designed and piloted by the Steering Group members. The survey will be created using QuestionPro ^20^ software and hosted on the Evidence Synthesis Ireland website. Participants will be asked to give their explicit consent to take part in the survey using yes/no questions. The survey will contain four open-ended questions. Three of the questions will focus on different stages of the rapid review process, and participants will be asked to answer as few or as many as they wish. The fourth question asks for any additional questions or comments on the rapid review process. The four questions are;

1. What questions or comments do you have about improving the process needed to plan a rapid review successfully?
2. What questions or comments do you have about improving how rapid reviews are carried out?
3. What questions or comments do you have about how the findings of rapid reviews are communicated to people?
4. Do you have any other questions or comments on how we plan, do and share the results of rapid reviews?

Participant demographic data will also be collected to monitor responses of different stakeholder groups and help refine and target the promotion of the survey towards under-represented groups if necessary. In line with previous methodology-focused PSPs ^17, 18^, the survey will be open for four weeks.

The survey will be advertised and distributed via the Steering Group and the PSP Partners. Specifically, members will distribute the survey via their networks, mailing lists, newsletters and social media. We do not have an *a priori* determined sample size and instead will distribute the survey widely with a view to reaching as wide an audience as possible. The survey and all other study materials can be found as Extended data ^21^.

***Data processing and verifying uncertainties (5 months- November 2020).*** The initial survey consultation process is expected to produce substantial ‘raw’ questions and comments indicating stakeholders’ areas of uncertainty. The survey data will be downloaded from QuestionPro ^20^. These raw questions will be categorised and refined by the National University of Ireland, Galway research team (CB, CH, DD) into summary questions that are clear, addressable by research, and understandable to all. Similar or duplicate responses will be combined where appropriate. Questions will be considered out of scope if they do not relate to planning, doing and sharing the results of rapid reviews within healthcare. We defined healthcare a priori as being related to the “treatment, control or prevention of disease, illness, injury or disability, and the care or aftercare of a person with these needs (whether or not the tasks involved have to be carried out by a health professional)” ^22^. Questions will also be considered out of scope if they are asking for information or advice, or being too broad, unclear, unrelated or off topic. Questions deemed to be out of scope will be compiled separately and made available for future use upon request.

The Steering Group will exercise oversight on data analysis and processing to ensure that the raw data are being interpreted appropriately. The summary questions will be developed in a reflective manner that is understandable to all audiences. The process will be conducted transparently to ensure that the finalised questions can be traced back to the raw response data.

Each in-scope question will be checked against existing sources of evidence to determine which questions remain unanswered. A question will be verified as unanswered if a synthesis gap is apparent following a search of all relevant systematic reviews published within the past three years. We judged a review to be systematic when it involved explicit methods to search, select, critically appraise and synthesise individual studies. If a systematic review has been conducted to answer any of the questions completely, the quality of that systematic review will be appraised using the AMSTAR 2 ^23^ tool to help inform decisions about the extent to which the question has been answered. If, following use of the AMSTAR 2 tool, a systematic review conducted to answer a question completely is identified as being of high quality (between 8-11), the question will be deemed as answered. Evidence checking will be completed by one researcher (CB) and verified by one other researcher (DD) Difference of opinion will be resolved by consultation with a third researcher if necessary.

Existing sources of evidence will be identified through a search of the PubMed bibliographic database for systematic reviews published from 2018 to the time of searching (2021) using a search strategy developed specifically for use in this project with the support of an experienced information specialist (see Table 1 for search strategy and limits applied). Due to limited resources (time and unavailability of translator) grey literature and publications not available in English will be excluded.

**Table 1.** Search strategy and limits (PubMed database).

Search strategy	((("Systematic Reviews as Topic" OR "Meta-Analysis as Topic" OR "Review Literature as Topic") AND "METHODS"[SH]) OR (("Syst Rev"[TA] OR "J Clin Epidemiol"[TA] OR "Res Synth Methods"[TA]) AND "METHODS"[SH]) OR (("Rapid Reviews" OR "systematic reviews" OR "systematic literature reviews") AND "METHODS"[SH] AND ("literature search*" OR Information Storage and Retrieval)) OR (("Rapid Reviews" OR "systematic reviews" OR "systematic literature reviews") AND "METHODS"[SH] AND ("plain language summar*" OR "lay summar*")) OR (("Rapid Reviews" OR "systematic reviews" OR "systematic literature reviews") AND "METHODS"[SH] AND “strength of evidence")) OR (("Rapid Reviews" OR "systematic reviews" OR "systematic literature reviews") "METHODS"[SH] AND "study selection")) OR (("Rapid Reviews" OR "systematic reviews" OR "systematic literature reviews") AND "METHODS"[SH] AND "data extraction")) OR (("Rapid Reviews" OR "systematic reviews" OR "systematic literature reviews") AND "METHODS"[SH] AND "quality assessment")) OR (("Rapid Reviews" OR "systematic reviews" OR "systematic literature reviews") AND "METHODS"[SH] AND "critical appraisal"))) NOT Protocol[TI])
Limit 1	Limited to ‘review’ and ‘systematic review’
Limit 2	Limited to three years prior to search taking place
Limit 3	Limited to English

*TA represents journal title abbreviation. SH represents subheading*

The JLA Question Verification Form, which describes the process used to verify question uncertainty, will be completed. In line with JLA guidance, the Question Verification Form includes details of the types and sources of evidence used to check uncertainty. This form will be published on the JLA website to enable stakeholders to understand how the PSP decided that these questions are unanswered and any limitations of this process.

The Steering Group will be asked to review and refine, as appropriate, the final list of summary questions for inclusion in the interim survey.

***Interim priority setting (1 month- April 2021).*** Interim priority setting is where the long list of questions is reduced to a shorter list that can be taken to the final priority setting workshop. This shortlist is aimed at a wide audience and will be administered using an electronic survey (QuestionPro software ^20^) and hosted on the Evidence Synthesis Ireland website. It will be designed and piloted by the Steering Group members. In this survey, stakeholders will be asked to prioritise summary questions developed from the initial survey in order of importance. This interim priority setting process will help reduce the long list to a short, manageable set of approximately 20 indicative questions that are clear, addressable by research and understandable. The survey will be open for four weeks and will be advertised and distributed like the initial survey via the Steering Group and the PSP Partners. Participant demographic data will be collected to monitor response rates from different stakeholder groups to help refine and target the promotion of the survey towards under-represented groups.

Questions prioritised in the interim survey will go forward for final prioritisation at a stakeholder workshop(s). Where the interim prioritisation does not produce a clear ranking or cut off point, the Steering Group will decide, and report, which questions are taken forward to the final prioritisation.

***Final prioritisation workshop (2 days- May 2021).*** The final priority setting stage will involve two half-day virtual workshops facilitated by the JLA to ensure transparency, accountability, fairness and appropriate representation. Based on the methods of the JLA, these workshops would ordinarily take place in person, but due to the Priority III study being conducted under restrictions contingent upon the global COVID-19 pandemic, the workshops will be held virtually. With input from the Steering Group, up to 24 contributors drawn from key stakeholder groups (patients, public, reviewers, researchers, clinicians, policymakers and funders) from a wide geographical spread, will be recruited to participate in a day of group discussion, plenary sessions and ranking exercises to determine the top 10 questions for research. Four virtual breakout rooms will be used to facilitate smaller group discussions. All participants will declare their interests, enabling a diverse group of stakeholders to exchange knowledge, perspectives, and experiences and inform the decision-making process. A maximum of four Steering Group members will be invited to participate in the workshops, with a steering group member in each breakout room to participate and answer any questions about the Priority III process. Additional Steering Group members who wish to attend will do so in an observer capacity. The outcome of the workshop will be consensus on a top 10 list of research priorities of unanswered questions about rapid review methodology.

***Disseminating findings.*** The results of the study will be disseminated through the JLA and Evidence Synthesis Ireland websites. The Steering Group will identify additional appropriate audiences to engage when sharing the results, such as researchers, funders, and patient and clinical communities. The steering group will also identify opportunities to collaborate and contribute evidence to answer the top 10 list of research priorities.

## Conclusion

The Priority III study will identify a top 10 of unanswered research questions regarding rapid review methodology, guided by an adaptation of the James Lind Alliance PSP process. The findings of the study will contribute to minimising research waste in rapid review methodology, ensuring that research resources will be used to answer questions on this topic that have been prioritised by key stakeholders internationally, including patient and the public, reviewers, researchers, clinicians, policymakers and funders. 

### Study status

At the time of submission, all stages up to the dissemination of findings had been completed. There are several factors that contributed to the submission of the protocol at this time:

1. The iterative nature of the PSP processes and the decisions that needed to be made by the Steering Group as the study progresses through to the later stages
2. Discussions with F1000/ HRB Open Research on affiliation of authors and the time dedicated to same

No results of the study have yet been disseminated.

## Data availability

### Underlying data

No data are associated with this article.

### Extended data

Open Science Framework: Priority III. https://doi.org/10.17605/OSF.IO/R6VFX ^21^.

This project contains the following extended data:

- Survey 1
  ○ Initial survey analysis Priority III.pdf (table used for analysis)
  ○ Initial survey download.pdf (survey)
  ○ Initial survey Priority III PIL.pdf (participant information leaflet)
  ○ Verification of summary questions.xlsx
- Survey 2
  ○ Interim survey analysis Priority III.pdf (table used for analysis)
  ○ Interim survey Priority-III-PIL.pdf (participant information leaflet)
- Workshop materials
  ○ Meeting consent form Priority III.pdf
  ○ Meeting PIL Priority III.pdf
  ○ Participant Worksheet.pdf

Data are available under the terms of the Creative Commons Attribution 4.0 International license (CC-BY 4.0).
