# Supplementary material for: What are the most important unanswered research questions on rapid review methodology? A James Lind Alliance research methodology Priority Setting Partnership: the Priority III study protocol
Source: HRB Open Res. 2021 Nov 18;4:80. Originally published 2021 Jul 23. [Version 2] doi: 10.12688/hrbopenres.13321.2 (PMC8506222; doi:10.12688/hrbopenres.13321.2)
Supplement: Peer review history

# Peer review: Reviewer response for version 2

**DOI:** 10.21956/hrbopenres.14671.r30855

**Published:** 2021-11-29

**Authors:** Michelle Haby

**Affiliations:** 1 Department of Chemical and Biological Sciences, Universidad de Sonora, Hermosillo, Sonora, Mexico; 2 Centre for Health Policy, Melbourne School of Population and Global Health, The University of Melbourne, Melbourne, Australia

I am satisfied that the authors have adequately responded to my concerns about the paper.

 In relation to their response to my second point about the use of AMSTAR 2: the authors clarified that they will use a rating of 8-11 to classify a systematic review as high quality but I am a little perplexed as to how this will work in practice as AMSTAR 2 has 16 items and is not intended to generate an overall score. However, I am happy for the authors to clarify this in any resulting publications of results.

Is the study design appropriate for the research question?

Yes

Is the rationale for, and objectives of, the study clearly described?

Yes

Are sufficient details of the methods provided to allow replication by others?

Partly

Are the datasets clearly presented in a useable and accessible format?

Yes

Reviewer Expertise:

I have experience in conducting both systematic reviews and rapid reviews. I have also been involved in the development and assessment of methodologies for rapid reviews.

I confirm that I have read this submission and believe that I have an appropriate level of expertise to confirm that it is of an acceptable scientific standard.

# Peer review: Reviewer response for version 1

**DOI:** 10.21956/hrbopenres.14503.r30324

**Published:** 2021-10-11

**Authors:** Yaolin Zhou

**Affiliations:** 1 Department of Pathology & Laboratory Medicine, Brody School of Medicine, East Carolina University, Greenville, NC, USA

Despite the increasing need and use of “rapid reviews” during the COVID-19 pandemic, the evidence behind these abbreviated reviews themselves, is rather scant. The authors, who are presumably members of the James Lind Alliance (JLA), propose a research prioritization study (Priority Setting Partnership), which consists of two stakeholder surveys, followed by a consensus workshop to identify the top 10 unanswered questions regarding rapid reviews.

 The steps described in the paper include:

1. Establishing a steering group (6 months) to oversee the PSP. The steering group includes members across stakeholder groups and are reasonable for the project activities.
2. Identifying and inviting potential partners (7 months). The authors state that there is no minimum or maximum number of partners. Patient and public partners will be reimbursed (the authors do not state how much). No minimum or maximum number of participants are identified.
3. Gathering uncertainties (1 month – Oct 2020) from patients and the public, reviewers, researchers, clinicians, policymakers and funders (by conducting an initial 4 question survey with all relevant stakeholders).
4. Data processing and verifying uncertainties (5 months) – raw questions will be categorized and refined into summary questions (downloaded from QuestionPro and then categorized and refined by a “NUI Galway” research team.
5. Interim priority setting ( 1 month – April 2021) – long list of questions is reduced to shorter list. Stakeholders will be asked to prioritize summary questions.
6. Final prioritization workshop (2 days – May 2021) – two half day virtual workshops of up to 24 key stakeholder members to participate in discussions (group discussion, plenary sessions, raking exercises, virtual breakout rooms). The outcome of the workshop will be consensus on a top 10 list of research priorities of unanswered questions about rapid review methodology.
7. Disseminating findings - results will be shared through JLA and Evidence Synthesis Ireland websites

Major Concern

- The paper is written in the future tense, but all steps except for 7 above, have already been completed per the authors. Throughout my reading, I am anticipating that this is a future study that has yet to be conducted. It comes as a surprise to me that at the very end, in the “study status,” the authors reveal all the steps except the final one (disseminating findings), have been completed.

Minor Concerns

- Minor grammatical error: *“Evidence suggests that rapid reviews are increasingly being commissioned, and the results used, to inform decision making by policymakers ….”* Please remove the comma after “results used.” The sentence should read: “Evidence suggests that rapid reviews are increasingly being commissioned, and the results used to inform decision making by policymakers…”
- Presumably the authors of the paper are on the steering group? This is not clearly stated.
- NUI Galway needs to be spelt out for readers unfamiliar that it represents National University of Ireland, Galway.
- The authors do not state how much the partners will be reimbursed.
- The time frames for the initial steps listed above are inconsistent. Some are written to only include the duration, but others include both duration (e.g., 1 month, 6 months, 2 days) and the month (Oct 2020, April 2021, May 2021). My recommendation would be to stick with one approach and be consistent (e.g., include the time period with actual dates because the duration can be implied).

Summary

 This paper describes a proposed research project that consists of a series of focus groups and stakeholder meetings to define the most critical 10 research questions regarding rapid reviews. It is well-written with minimal grammatical and syntax errors, but I am unclear why it was written in the future tense. The authors describe a proposed study, when in fact, all steps of the project have been conducted except for the last one. When I get to the final section of the paper and realize the proposed steps have been completed, my natural questions include; How did the sessions go? Who was recruited to participate? How many were recruited and what was the breakdown? How much were the participants reimbursed for their time? What did the questions reveal? What are the next steps? But these questions are unanswered by the authors because the paper is written as a proposed project, despite the steps having already been completed. This issue needs to be resolved before I am fully comfortable with accepting in the current state.

Recommendation

 Thank you for the opportunity to review this paper. My recommendation would be to convert this into a dissemination paper, such as the PRioRiTy (Prioritising Recruitment in Randomised Trials) publications the authors cite (references 17 and 18). If one of the purposes of the project is to conduct research on the top 10 list of research priorities of unanswered questions about rapid review methodologies AND then disseminate those findings, then the authors may want to consider converting their paper into one that serves the latter purpose.

Is the study design appropriate for the research question?

Yes

Is the rationale for, and objectives of, the study clearly described?

Yes

Are sufficient details of the methods provided to allow replication by others?

Yes

Are the datasets clearly presented in a useable and accessible format?

Not applicable

Reviewer Expertise:

Molecular diagnostics, health policy, diagnostic testing, healthcare value, test utilization

I confirm that I have read this submission and believe that I have an appropriate level of expertise to confirm that it is of an acceptable scientific standard, however I have significant reservations, as outlined above.

# Response

**Published:** 2021-11-05

**Authors:** Claire Beecher

**Affiliations:** HRB-TMRN & ESI, Ireland

***Reviewer Comment 1***

 The paper is written in the future tense, but all steps except for 7 above, have already been completed per the authors. Throughout my reading, I am anticipating that this is a future study that has yet to be conducted. It comes as a surprise to me that at the very end, in the “study status,” the authors reveal all the steps except the final one (disseminating findings), have been completed.

***Response 1***

 Thank you for raising this point. This protocol paper gives much more detail on the methods than will be contained in the results paper hence the importance of this protocol paper.

 There are several factors that contributed to the submission of the protocol at this time:

1. The evolving nature of the PSP process and the decisions that needed to be made by the steering group as the study progresses through to the later stages.
2. Discussions with F1000/ HRB Open Research on affiliation of public partner authors and the time to agree same.
3. Attempts were made to be submit the protocol prior to the completion of the workshop but due to an issue with submission process, this was not possible until after the workshop had been completed (issue rectified by HRB Open Research).

Although the protocol would ideally have been submitted at an earlier stage, this was not possible given the above factors.

 We have retained future tense in keeping with protocols papers but have added the following to the paper to be more transparent:

 'This protocol was submitted prior to the consensus workshops and published after completion of the final prioritisation workshop. There are several factors that contributed to the late submission of the protocol. We had to balance the evolving nature of the PSP processes and having sufficient clarity on processes for inclusion in a protocol. We also encountered delays due to useful discussions with F1000/ HRB Open Research on affiliation of public partner authors and technical problems with submission attempts prior to the consensus workshop’ (4 ^th^ paragraph under heading ‘James Lind Alliance PSP process’)

***Reviewer Comment 2***

 Minor grammatical error: “Evidence suggests that rapid reviews are increasingly being commissioned, and the results used, to inform decision making by policymakers ….” Please remove the comma after “results used.” The sentence should read: “Evidence suggests that rapid reviews are increasingly being commissioned, and the results used to inform decision making by policymakers…”

***Response 2***

 Thank you very much for highlighting this, it has now been amended.

***Reviewer Comment 3***

 Presumably the authors of the paper are on the steering group? This is not clearly stated.

***Response 3***

 Not all co-authors are members of the steering group. All co-authors have been included based on their contribution as identified using the CREDiT Taxonomy.

***Reviewer Comment 4***

 NUI Galway needs to be spelt out for readers unfamiliar that it represents National University of Ireland, Galway.

***Response 4***

 Thank you very much, this has now been updated.

***Reviewer Comment 5***

 The authors do not state how much the partners will be reimbursed.

***Response 5***

 This has not been stated as there is no reimbursement for partners.

***Reviewer Comment 6***

 The time frames for the initial steps listed above are inconsistent. Some are written to only include the duration, but others include both duration (e.g., 1 month, 6 months, 2 days) and the month (Oct 2020, April 2021, May 2021). My recommendation would be to stick with one approach and be consistent (e.g., include the time period with actual dates because the duration can be implied).

***Response 6***

 Thank you for pointing this out - this has now been updated as suggested (please note some overlap in timeline included between establishing steering group and identifying and inviting partners as these processes ran concurrently for a limited time).

***Reviewer Comment 7***

 This paper describes a proposed research project that consists of a series of focus groups and stakeholder meetings to define the most critical 10 research questions regarding rapid reviews. It is well-written with minimal grammatical and syntax errors, but I am unclear why it was written in the future tense. The authors describe a proposed study, when in fact, all steps of the project have been conducted except for the last one. When I get to the final section of the paper and realize the proposed steps have been completed, my natural questions include; How did the sessions go? Who was recruited to participate? How many were recruited and what was the breakdown? How much were the participants reimbursed for their time? What did the questions reveal? What are the next steps? But these questions are unanswered by the authors because the paper is written as a proposed project, despite the steps having already been completed. This issue needs to be resolved before I am fully comfortable with accepting in the current state.

***Response 7***

 Thank you for raising this feedback. Response to the point has been provided in point 1.

***Reviewer Comment 8***

 Thank you for the opportunity to review this paper. My recommendation would be to convert this into a dissemination paper, such as the PRioRiTy (Prioritising Recruitment in Randomised Trials) publications the authors cite (references 17 and 18). If one of the purposes of the project is to conduct research on the top 10 list of research priorities of unanswered questions about rapid review methodologies AND then disseminate those findings, then the authors may want to consider converting their paper into one that serves the latter purpose

***Response 8***

 Thank you very much for your time reviewing this protocol. We do take on board your feedback and although ideally the protocol would have been submitted at an earlier stage, this was not possible given the factors that have been listed above.

 This protocol paper gives much more detail on the methods than will be contained in the results paper hence the importance of this protocol paper. We have retained future tense in keeping with protocols papers but have added the following to the paper to be more transparent:

 'This protocol was submitted prior to the consensus workshops and published after completion of the final prioritisation workshop. There are several factors that contributed to the late submission of the protocol. We had to balance the evolving nature of the PSP processes and having sufficient clarity on processes for inclusion in a protocol. We also encountered delays due to useful discussions with F1000/ HRB Open Research on affiliation of public partner authors and technical problems with submission attempts prior to the consensus workshop’ (4 ^th^ paragraph under heading ‘James Lind Alliance PSP process’)

# Peer review: Reviewer response for version 1

**DOI:** 10.21956/hrbopenres.14503.r30327

**Published:** 2021-10-11

**Authors:** Michelle Haby

**Affiliations:** 1 Department of Chemical and Biological Sciences, Universidad de Sonora, Hermosillo, Sonora, Mexico; 2 Centre for Health Policy, Melbourne School of Population and Global Health, The University of Melbourne, Melbourne, Australia

This is a very interesting and valuable project. It has a well-described and well written methodology for priority setting. The results will provide an important contribution to the field. I do have some comments, however, for consideration:

 I was rather surprised to reach the end of the protocol and find out that the research had already been completed, except for the dissemination of the findings. It made me wonder what then the purpose of peer review was, except perhaps to ensure accuracy of reporting. While I imagine that there are good reasons for the protocol being submitted at this late stage, I think it would be more transparent (and helpful for the reader) that this is made clear from the beginning, along with the reason. Thus, I suggest that the protocol should be written in the past tense, where applicable, and dates added for all stages.

 The inclusion of the process for checking that questions were indeed still unanswered is a very valuable inclusion in the methods – I look forward to seeing the results of this process. I hope that the authors can include in the publication of the results the questions that are already answered and the sources of the existing evidence. I do have some concerns about the methodology here and suggest that the authors include a justification of their choice of methods or further clarification:

- The quality of the systematic reviews will be appraised using AMSTAR 2 but there is no description in the protocol as to how the score will be used in deciding if the question has been answered, e.g. does it need to have a minimum quality?
- Only PubMed will be searched and only reviews published in English will be included. It is not clear how many reviewers will be involved in the quality assessment and why non-English and grey literature is not being included. If the authors were to evaluate their own review process using AMSTAR 2 I expect that it would not score highly. Thus, the authors should provide greater clarity and a justification for their choice of methods for this part of the protocol.

In the paragraph headed ‘Data processing and verifying uncertainties (5 months)’ I noticed that the scope of the priority setting exercise is limited to the healthcare setting. This is a very important limitation but no explanation is given for why. Why not include public health or health system questions, which are equally (if not more) important for rapid reviews?

 It is not clear from the protocol if there was any attempt to include the views of people from low- to middle-income countries, which may be able to offer a broader perspective of the unanswered questions. Can this be clarified?

 When defining rapid reviews (in the abstract and introduction) I suggest noting that an important distinction from systematic reviews is that rapid reviews are generally conducted with the needs of decision-makers in mind – see for example:

- Hartling L, Guise JM, Kato E, Anderson J, Aronson N, Belinson S, et al. EPC Methods: An Exploration of Methods and Context for the Production of Rapid Reviews. Research White Paper. Prepared by the Scientific Resource Center under Contract No. 290-2012-00004-C. AHRQ Publication No. 15-EHC008-EF. Rockville, MD: Agency for Healthcare Research and Quality; 2015 ^1^.
- Haby MM, Chapman E, Clark R, Barreto J, Reveiz L, Lavis JN. What are the best methodologies for rapid reviews of the research evidence for evidence-informed decision making in health policy and practice: a rapid review. Health Res Policy Syst. 2016;14(1):83 ^2^ (my article).

Minor comments:

- Third paragraph of introduction: 1) in the first sentence it is not clear what is ‘often poorly reported’, and 2) the last sentence doesn’t make sense to me. Consider revising both.
- Under ‘Gathering uncertainties…’ – ‘content’ instead of ‘consent’ is used.

Is the study design appropriate for the research question?

Yes

Is the rationale for, and objectives of, the study clearly described?

Yes

Are sufficient details of the methods provided to allow replication by others?

Partly

Are the datasets clearly presented in a useable and accessible format?

Yes

Reviewer Expertise:

I have experience in conducting both systematic reviews and rapid reviews. I have also been involved in the development and assessment of methodologies for rapid reviews.

I confirm that I have read this submission and believe that I have an appropriate level of expertise to confirm that it is of an acceptable scientific standard, however I have significant reservations, as outlined above.

## References

[1] : EPC Methods: An Exploration of Methods and Context for the Production of Rapid Reviews. 25654160

[2] : What are the best methodologies for rapid reviews of the research evidence for evidence-informed decision making in health policy and practice: a rapid review. *Health Res Policy Syst* .2016;14(1) : 10.1186/s12961-016-0155-7 83 10.1186/s12961-016-0155-7 27884208PMC5123411

# Response

**Published:** 2021-11-05

**Authors:** Claire Beecher

**Affiliations:** HRB-TMRN & ESI, Ireland

***Reviewer Comment 1***

 I was rather surprised to reach the end of the protocol and find out that the research had already been completed, except for the dissemination of the findings. It made me wonder what then the purpose of peer review was, except perhaps to ensure accuracy of reporting. While I imagine that there are good reasons for the protocol being submitted at this late stage, I think it would be more transparent (and helpful for the reader) that this is made clear from the beginning, along with the reason. Thus, I suggest that the protocol should be written in the past tense, where applicable, and dates added for all stages.

***Response 1***

 Thank you for raising this point. This protocol paper gives much more detail on the methods than will be contained in the results paper hence the importance of this protocol paper.

 There are several factors that contributed to the submission of the protocol at this time:

1. The evolving nature of the PSP process and the decisions that needed to be made by the steering group as the study progresses through to the later stages.
2. Discussions with F1000/ HRB Open Research on affiliation of public partner authors and the time to agree same.
3. Attempts were made to be submit the protocol prior to the completion of the workshop but due to an issue with submission process, this was not possible until after the workshop had been completed (issue rectified by HRB Open Research).

Although the protocol would ideally have been submitted at an earlier stage, this was not possible given the above factors.

 We have retained future tense in keeping with protocols papers but have added the following to the paper to be more transparent:

 'This protocol was submitted prior to the consensus workshops and published after completion of the final prioritisation workshop. There are several factors that contributed to the late submission of the protocol. We had to balance the evolving nature of the PSP processes and having sufficient clarity on processes for inclusion in a protocol. We also encountered delays due to useful discussions with F1000/ HRB Open Research on affiliation of public partner authors and technical problems with submission attempts prior to the consensus workshop’ (4 ^th^ paragraph under heading ‘James Lind Alliance PSP process’)

***Reviewer Comment 2***

 The inclusion of the process for checking that questions were indeed still unanswered is a very valuable inclusion in the methods – I look forward to seeing the results of this process. I hope that the authors can include in the publication of the results the questions that are already answered and the sources of the existing evidence. I do have some concerns about the methodology here and suggest that the authors include a justification of their choice of methods or further clarification:

- The quality of the systematic reviews will be appraised using AMSTAR 2 but there is no description in the protocol as to how the score will be used in deciding if the question has been answered, e.g. does it need to have a minimum quality?
- Only PubMed will be searched and only reviews published in English will be included. It is not clear how many reviewers will be involved in the quality assessment and why non-English and grey literature is not being included. If the authors were to evaluate their own review process using AMSTAR 2 I expect that it would not score highly. Thus, the authors should provide greater clarity and a justification for their choice of methods for this part of the protocol.

***Response 2***

 Thank you for this comment. Yes, in the final paper all results and original documents used will be presented via a link to Open Science Framework to ensure transparency of this process.

- The following text has been included in relation to how the AMSTAR 2 score would be used in deciding if a question is answered: ‘If, following use of the AMSTAR 2 tool, a systematic review conducted to answer any of the questions completely is identified as being of high quality (between 8-11), the question will be deemed as answered.’ (3 ^rd ^paragraph under heading ‘Data processing and verifying uncertainties’).

- It is stated that “Evidence checking will be completed by one researcher (CB) and verified by one other researcher (DD) Difference of opinion will be resolved by consultation with a third researcher if necessary” (3 ^rd ^paragraph under heading ‘Data processing and verifying uncertainties’).

Thank you very much for highlighting this omission. A rationale for non-English and grey literature not being included has been added to the text (4 ^th ^paragraph under heading ‘Data processing and verifying uncertainties’).

***Reviewer Comment 3***

 In the paragraph headed ‘Data processing and verifying uncertainties (5 months)’ I noticed that the scope of the priority setting exercise is limited to the healthcare setting. This is a very important limitation but no explanation is given for why. Why not include public health or health system questions, which are equally (if not more) important for rapid reviews?

***Response 3***

 We wish to clarify that we had a relatively broad definition of healthcare which we defined a priori as being;

 “treatment, control or prevention of disease, illness, injury or disability, and the care or aftercare of a person with these needs (whether or not the tasks involved have to be carried out by a health professional)”. This definition has been adapted from the UK Department of Health and Social Care guidance document titled ‘National Framework for NHS Continuing Healthcare and NHS-funded Nursing Care’.

 We have added this to the paper (1 ^st ^paragraph under heading ‘Data processing and verifying uncertainties’).

***Reviewer Comment 4***

 It is not clear from the protocol if there was any attempt to include the views of people from low- to middle-income countries, which may be able to offer a broader perspective of the unanswered questions. Can this be clarified?

***Response 4***

 The views of people from low- to middle-income countries are very important in each stage of the Priorty III stages.

 Reference to the importance of representation from underrepresented groups has been highlighted by the statement “Participant demographic data will also be collected to monitor responses of different stakeholder groups and help refine and target the promotion of the survey towards under-represented groups if necessary” within the gathering uncertainties and interim priority setting headings/ stages.

 The final prioritisation workshop stage has been updated to highlight that workshop participants will be drawn from a wide geographical spread including representation from low-middle income countries.

***Reviewer Comment 5***

 When defining rapid reviews (in the abstract and introduction) I suggest noting that an important distinction from systematic reviews is that rapid reviews are generally conducted with the needs of decision-makers in mind – see for example:

- Hartling L, Guise JM, Kato E, Anderson J, Aronson N, Belinson S, et al. EPC Methods: An Exploration of Methods and Context for the Production of Rapid Reviews. Research White Paper. Prepared by the Scientific Resource Center under Contract No. 290-2012-00004-C. AHRQ Publication No. 15-EHC008-EF. Rockville, MD: Agency for Healthcare Research and Quality; 20151.

- Haby MM, Chapman E, Clark R, Barreto J, Reveiz L, Lavis JN. What are the best methodologies for rapid reviews of the research evidence for evidence-informed decision making in health policy and practice: a rapid review. Health Res Policy Syst. 2016;14(1):832 (my article).

***Response 5***

 Thank you for this feedback, however we do feel that the point you have raised is addressed in the first two paragraphs of the introduction. For example, we say (paragraph 1 & 2 of introduction),

 “While full systematic reviews can, and have been, completed rapidly, rapid reviews have emerged more commonly as a form of evidence synthesis in which certain steps of the systematic review process are omitted or simplified to produce information more efficiently within limited resources to inform healthcare decisions.

 Evidence suggests that rapid reviews are increasingly being commissioned, and the results used to inform decision making by policymakers and funders as a more resource efficient alternative to conventional systematic reviews…..”

***Reviewer Comment 6***

 Third paragraph of introduction: 1) in the first sentence it is not clear what is ‘often poorly reported’, and 2) the last sentence doesn’t make sense to me. Consider revising both.

***Response 6***

 Thank you very much for this feedback- both sentences have been updated (paragraph 3 of introduction)

***Reviewer Comment 7***

 Under ‘Gathering uncertainties…’ – ‘content’ instead of ‘consent’ is used.

***Response 7***

 Thank you very much for highlighting this, it has now been amended (paragraph 1 under heading ‘Gathering uncertainties’)

# Peer review: Reviewer response for version 1

**DOI:** 10.21956/hrbopenres.14503.r30325

**Published:** 2021-10-01

**Authors:** Timothy O'Leary

**Affiliations:** 1 Veterans Health Administration, Washington, DC, USA; 2 University of Maryland School of Medicine, Baltimore, MD, USA

The paper provides a well-structured and readable overview of a project intended to develop a better understanding of the strengths and limitations of “rapid reviews,” and presumably provide a framework for better standardizing this form of evidence synthesis.

 I note that according to the authors “ *At the time of submission, all stages up to the dissemination of findings had been completed*.”

 Normally I would expect the material in the current paper to be included in the final publication, rather than in a separate protocol paper that seems to have been written essentially contemporaneously with the final work.  I will not second-guess this decision, but will instead comment as if the project were not now largely complete.

 There are a few places where I believe that the reader’s understanding of the project could be improved by including either tables or supplementary material to improve the specificity of the document.  The document states that:

*"Membership of the Steering Group will include individuals and representatives from organisations, including patients and the public, reviewers, researchers, clinicians, policymakers, and funders. The Steering Group will comprise of up 25 members across stakeholder groups."*

 Either the membership of this Steering group should be provided, or the types of groups and organizations that were contacted to participate should be provided. Were these groups restricted to Ireland, or to Europe, or to English speaking countries, or to advanced economies?  It seems likely that the ultimate product coming from a Eurocentric steering committee will be different than that which involves individuals from Asia, Africa, and South America. On the other hand, it would be difficult to adequately represent researchers, clinicians, policymakers, and funders across a wide geographic distribution.   

 The areas of expertise associated with steering group members (statistics, survey methodology, etc.) should be described in more detail.

 Similarly, the nature of the “partners”  should also be better defined, as should a bit about the total number of stakeholders expected to participate.  The final report should include information on the nature and number of stakeholders that were contacted and who declined to participate.

Is the study design appropriate for the research question?

Yes

Is the rationale for, and objectives of, the study clearly described?

Yes

Are sufficient details of the methods provided to allow replication by others?

No

Are the datasets clearly presented in a useable and accessible format?

Not applicable

Reviewer Expertise:

I currently perform systematic reviews and meta-analysis for covid19-related diagnostics.  I have previous experience in randomized clinical trials, genomic epidemiology, and preclinical research.

I confirm that I have read this submission and believe that I have an appropriate level of expertise to confirm that it is of an acceptable scientific standard, however I have significant reservations, as outlined above.

# Response

**Published:** 2021-11-05

**Authors:** Claire Beecher

**Affiliations:** HRB-TMRN & ESI, Ireland

***Reviewer Comment 1***

 I note that according to the authors “At the time of submission, all stages up to the dissemination of findings had been completed.”

 Normally I would expect the material in the current paper to be included in the final publication, rather than in a separate protocol paper that seems to have been written essentially contemporaneously with the final work. I will not second-guess this decision, but will instead comment as if the project were not now largely complete.

***Response 1***

 Thank you for raising this point. This protocol paper gives much more detail on the methods than will be contained in the results paper hence the importance of this protocol paper.

 There are several factors that contributed to the submission of the protocol at this time:

1. The evolving nature of the PSP process and the decisions that needed to be made by the steering group as the study progresses through to the later stages.
2. Discussions with F1000/ HRB Open Research on affiliation of public partner authors and the time to agree same.
3. Attempts were made to be submit the protocol prior to the completion of the workshop but due to an issue with submission process, this was not possible until after the workshop had been completed (issue rectified by HRB Open Research).

Although the protocol would ideally have been submitted at an earlier stage, this was not possible given the above factors.

 We have retained future tense in keeping with protocols papers but have added the following to the paper to be more transparent:

 'This protocol was submitted prior to the consensus workshops and published after completion of the final prioritisation workshop. There are several factors that contributed to the late submission of the protocol. We had to balance the evolving nature of the PSP processes and having sufficient clarity on processes for inclusion in a protocol. We also encountered delays due to useful discussions with F1000/ HRB Open Research on affiliation of public partner authors and technical problems with submission attempts prior to the consensus workshop’ (4 ^th^ paragraph under heading ‘James Lind Alliance PSP process’)

***Reviewer Comment 2***

 There are a few places where I believe that the reader’s understanding of the project could be improved by including either tables or supplementary material to improve the specificity of the document. The document states that:

 "Membership of the Steering Group will include individuals and representatives from organisations, including patients and the public, reviewers, researchers, clinicians, policymakers, and funders. The Steering Group will comprise of up 25 members across stakeholder groups."

 Either the membership of this Steering group should be provided, or the types of groups and organizations that were contacted to participate should be provided. Were these groups restricted to Ireland, or to Europe, or to English speaking countries, or to advanced economies? It seems likely that the ultimate product coming from a Eurocentric steering committee will be different than that which involves individuals from Asia, Africa, and South America. On the other hand, it would be difficult to adequately represent researchers, clinicians, policymakers, and funders across a wide geographic distribution

***Response 2***

 We do understand the relevance of the membership of the steering group; however we haven’t included detailed information on the steering group here as information on the areas of expertise associated with steering group members will be described in detail in the results paper that presents the outcome of each of the seven stages of the priority setting process.

 Thank you for raising this point- we confirm that the steering group members are drawn from a wide geographical spread- this has now been highlighted within the text (2 ^nd^ paragraph under heading ‘Establishing the Steering Group’). Given the international relevance of the topic, as you have highlighted, it is very important that Priority III steering group members were not restricted to Ireland, Europe, English speaking countries, or advanced economies.

***Reviewer Comment 3***

 The areas of expertise associated with steering group members (statistics, survey methodology, etc.) should be described in more detail.

***Response 3***

 Thank you very much for suggesting. We haven’t included detailed information on the steering group here as information on the areas of expertise associated with steering group members will be described in detail in the results paper that presents the outcome of each of the seven stages of the priority setting process.

***Reviewer Comment 4***

 Similarly, the nature of the “partners” should also be better defined, as should a bit about the total number of stakeholders expected to participate. The final report should include information on the nature and number of stakeholders that were contacted and who declined to participate

***Response 4***

 Thank you very much for suggesting this. We have now added further information on the nature of partners (first line under heading ‘Identifying and inviting potential partners’).

 We agree fully that the final report should include information on the nature and number of stakeholders that were contacted and who declined to participate.

# Peer review: Reviewer response for version 1

**DOI:** 10.21956/hrbopenres.14503.r30257

**Published:** 2021-09-23

**Authors:** Kristina Staley

**Affiliations:** 1 TwoCan Associates, Ross-on-Wye, UK

This is a clear summary of the approach taken to identify priorities for research on rapid reviews. It is consistent with the standard JLA approach and is well-described. I do not have any concerns about this. I’m not an expert on rapid reviews, so I can’t comment on that.

 I am interested in specifics about how well the involvement works, what kind of questions come forward and how possible tensions may be resolved between different stakeholders' views on what's important. But as no results have yet been reported, these questions are not yet relevant.

Is the study design appropriate for the research question?

Yes

Is the rationale for, and objectives of, the study clearly described?

Yes

Are sufficient details of the methods provided to allow replication by others?

Yes

Are the datasets clearly presented in a useable and accessible format?

Not applicable

Reviewer Expertise:

Patient and public involvement in research

I confirm that I have read this submission and believe that I have an appropriate level of expertise to confirm that it is of an acceptable scientific standard.
